# Prognostic significance of nomograms integrating IL‐37 expression, neutrophil level, and MMR status in patients with colorectal cancer

**DOI:** 10.1002/cam4.1663

**Published:** 2018-07-13

**Authors:** Bing Zhu, Jie Luo, Yiyao Jiang, Luhua Yu, Mulin Liu, Jun Fu

**Affiliations:** ^1^ Department of Gastrointestinal Surgery The first Affiliated Hospital of Bengbu Medical College Bengbu China; ^2^ Department of Cardiovascular Surgery Tianjin First Central Hospital Tianjin China; ^3^ Department of Otolaryngology‐Head and Neck Surgery The first Affiliated Hospital of Bengbu Medical College Bengbu China

**Keywords:** colorectal cancer, IL‐37, neutrophils

## Abstract

Interleukin (IL)‐37 and neutrophils are considered to be involved in human cancer, but their prognostic significance in colorectal cancer (CRC) has not been elucidated. The aim of this study was to evaluate the prognostic value of IL‐37 expression and neutrophil levels in CRC. We retrospectively analyzed IL‐37 expression, CD66b^+^ neutrophil levels, and mismatch repair (MMR) status in 337 paraffin‐embedded CRC specimens from the training cohort by immunohistochemistry. Their prognostic values were assessed using Kaplan‐Meier curves and multivariate Cox regression models. Moreover, several risk factors were used to form nomograms to evaluate survival, and the performance of the nomograms was assessed with respect to calibration, discrimination, and clinical usefulness. Further validation was performed in an independent cohort of 245 cases. Low IL‐37 expression and high CD66b^+^ neutrophil levels were significantly associated with diminished disease‐free survival (DFS) and overall survival (OS), and patients with MMR‐deficient CRC had better clinical outcomes. Furthermore, multivariate Cox analysis identified IL‐37, CD66b^+^ neutrophils, and MMR status as independent prognostic factors for DFS and OS. Two nomograms integrating the three markers with four clinicopathological risk factors were developed and validated for predicting DFS and OS with good calibration and discrimination (C‐index: training cohort, 0.798 (95% confidence interval:0.764‐0.832) and 0.828 (0.796‐0.860), respectively; validation cohort, 0.739 (0.696‐0.783) and 0.761 (0.715‐0.808), respectively). Decision curve analysis demonstrated that the nomograms were clinically useful. Intratumoral IL‐37, CD66b^+^ neutrophils, and MMR status were independent prognostic factors for CRC patients. Nomograms incorporating these biomarkers and clinicopathological features could be conveniently used to facilitate the individualized prediction of DFS and OS.

## 1 INTRODUCTION

Colorectal cancer (CRC) is one of the most common malignancies and accounts for a significant number of cancer‐related deaths worldwide.^1^ Although outcome and the need for adjuvant chemotherapy are routinely determined using the International Union Against Cancer (UICC) Tumor‐Node‐Metastasis (TNM) staging system, large variations in prognosis have been showed among patients with disease of the same stage and undergoing similar treatment.^2^, ^3^, ^4^ In fact, other tumor‐associated characteristics, such as those intrinsic to tumor cells and those related to the interactions of cancer cells with microenvironmental stimuli, might similarly influence CRC prognosis and be applied to evaluate the necessity of further treatment.^5^, ^6^

Interleukin (IL)‐37 is a new member of the IL‐1 family that encompasses 11 structurally related members sharing a β‐barrel motif.^7^ Nevertheless, entirely distinct with most IL‐1 family members, that are characterized with proinflammatory functions, IL‐37 has emerged as an important inhibitor of the innate immune response. With the accumulated evidence, IL‐37 has been recognized as a typical anti‐inflammatory cytokine related to autoimmune diseases, endotoxemia, inflammatory liver injury, obesity, and cancer.^8^, ^9^, ^10^, ^11^, ^12^ Gao et al^9^ found that the intratumoral injection of IL‐37 inhibited tumor growth and the antitumor activity of IL‐37 was abrogated in mice models. Moreover, the expression of IL‐37 was decreased in tumor tissues of hepatocellular carcinoma patients, and the expression level was negatively correlated with tumor size.^13^ Also, high levels of IL‐37 were associated with improved overall survival and disease‐free survival.^13^, ^14^, ^15^ Furthermore, the antitumor activity was found by recruiting more NK cells in hepatocellular carcinoma and inhibiting tumor angiogenesis in nonsmall cell lung cancer.^13^, ^14^ Recent study by Zhao found that IL‐37 inhibited colon tumor formation in the mice model and sensitized the cancer cell to chemotherapy drugs.^15^ This suggests that IL‐37 could play an important role in tumor growth and may be useful for chemotherapy and tumor immunotherapy. Despite this knowledge, the molecular and prognostic functions of IL‐37 in CRC are not yet conclusive.

Neutrophils are the predominant circulating granulocyte in humans and comprise 50%‐75% of circulating leukocytes. Extensive literature has suggested that the tumor‐associated neutrophils (TANs) are of clinical importance in cancer 16, 17, 18, 19 Increased infiltration of neutrophils has been observed in many types of human cancers.^18^, ^19^ Growing evidence shows that TANs play important roles in cancer development, progression, and resistance to therapy.^17^, ^18^ A meta‐analysis of 3946 patients from 20 studies concluded that neutrophils are typically protumor and are strongly associated with poorer prognosis in the majority of human tumors.^20^ However, under different tumor microenvironments, neutrophils can be polarized into either an antitumor (N1) or protumor (N2) phenotype and show different functions.^18^, ^21^ Plasticity in neutrophils has recently been shown to lead to both pro‐ and antitumor effects.^18^ Therefore, the role of TANs in tumors remains controversial, including CRC.^18^, ^19^, ^22^ Many studies have found neutrophils to be an independent factor of poor prognosis and promote the growth of distance metastases in CRC.^23^, ^24^ However, other studies have revealed that tumor‐infiltrating neutrophils are associated with a better prognosis in CRC and might promote antitumor immunity.^22^, ^25^ CD66b was found to be a reliable marker to identify TANs in cancer tissues using immunohistochemistry, including in CRC tissues.^17^, ^19^, ^22^, ^24^, ^26^ Thus, the prognostic role of TANs in CRC is not yet conclusive, and the relationship of IL‐37 and CD66b^+^ TANs has not been previously elucidated.

Division of CRCs into molecular subsets yields important consequences for prognosis and therapeutic response.^27^, ^28^, ^29^ The microsatellite instability (MSI) immune subgroup, accounting for 15% of all CRCs, are a result of deficient cellular DNA mismatch repair (dMMR) mechanisms.^28^ Deficient MMR can result from a germline mutation in an MMR gene (MLH1, MSH2, MSH6, PMS2), that is, Lynch syndrome (LS). dMMR CRCs are notable for greater survivability, particularly in Stage II/III CRC.^28^, ^30^, ^31^ Retrospective studies have demonstrated improved survival in patients with MMR‐competent tumors after receiving 5‐FU‐based chemotherapy.^32^, ^33^ A large multicenter study supports the use of adjuvant chemotherapy with fluoropyrimidine plus oxaliplatin for stage III‐dMMR colon cancer.^34^ The extent of neutrophil infiltration in human CRC has been shown to differ based on MMR status.^6^, ^33^

In the study, we explored IL‐37 expression in CRC patients by immunohistochemistry (IHC) and explored its associations with clinicopathological factors and outcomes. We also investigated the prognostic value of CD66b^+^ TANs and MMR status and the interrelationship of IL‐37, CD66b^+^TANs, and MMR status. Moreover, we developed two predictive nomograms integrating IL‐37, CD66b^+^ TANs, MMR status, carcinoembryonic antigen (CEA), depth of invasive, lymph node metastasis, and distant metastasis to evaluate individualized disease‐free survival (DFS) and overall survival (OS).

## 2 MATERIALS AND METHODS

### 2.1 Patients and specimens

In the study, two cohorts with a total of 582 formalin‐fixed paraffin‐embedded (FFPE) specimens from the 582 patients with CRC were included. In the training cohort, data were collected from 337 patients with incident, primary, biopsy‐confirmed CRC diagnosed between January 2006 and December 2007 at the First Affiliated Hospital of Bengbu Medical College, Bengbu, China. The inclusion criteria included the availability of hematoxylin‐ and eosin‐stained slides with invasive tumor components, no history of treated cancer, the availability of clinicopathological characteristics, and the availability of follow‐up data. We included another 245 patients with the same criteria as above, as the validation cohort, between January 2008 and December 2009. And all these patients with metastatic CRC included in this study had undergone radical resection of both the primary and metastatic sites. All patients were staged again according to the eighth edition of the Tumor‐Node‐Metastasis (TNM) staging system. Two independent pathologists reassessed all these samples. Follow‐up information was obtained from hospital records for patients who were lost to follow‐up. The duration of follow‐up was calculated from the time of surgery to the last follow‐up date, and information of the survival status at the last follow‐up was also collected. The study was approved by the Research Ethics Committee of the First Affiliated Hospital of Bengbu Medical College, and informed consent was obtained from all participants.

### 2.2 Immunohistochemical staining

Formalin‐fixed paraffin‐embedded samples were processed for IHC, as previously described.^17^, ^19^, ^35^ Sections were cut at a thickness of 3 μm, de‐waxed in xylene, and rehydrated in decreasing concentrations of ethanol. Before staining, the sections were subjected to endogenous peroxidase blocking for 10 minutes in 1% H_2_O_2_ solution in methanol and then heated in a microwave in 10 mmol/L citrate buffer, pH 6.0 for 30 minutes. Serum blocking was performed for 30 minutes using 10% normal rabbit serum. The slides were incubated in primary antibody overnight at 4°C at a concentration of 1:1000 for IL‐37 (ab57187, Abcam, Cambridge, MA, USA), 1:200 for CD66b (Clone G10F5, BD Biosciences), 1:100 for MLH1and MSH6, and 1:50 for MSH2 and PMS2 (product codes: M3640, M3646, M3639 and M3647, respectively; Dako UK Ltd, Cambridge shire, UK), and then incubated with a labeled polymer/HRP amplification system (EnVision^™^, Dako Cytomation, Denmark) for 30 minutes. Finally, after 3, 3′‐diaminobenzidinetetrahydrochloride (DAB) was used to visualize the signal development, the sections were counter stained with 20% hematoxylin.

### 2.3 Evaluation of immunohistochemical staining

Analysis was performed by two independent pathologists in a blinded manner. Tissue sections were screened at a low power (100×), and five representative fields were selected at a high power (200×) with Leica DM IRB inverted research microscope (Leica Microsystems, Wetzlar, Germany). All discrepancies were resolved by a joint review of the slides in question.

IL‐37 density was quantified according to the percentage of positively stained cells and the staining intensity. The staining of each specimen was evaluated using a semiquantitative H score, which was calculated by multiplying the result of a 4‐stepscale (0 = negative, 0.5 = weak staining, 1 = moderate staining, 1.5 = strong staining) and the fraction of positively stained cells from 0% to 100%;the H score ranged from 0 to 150.^36^ For IL‐37, the H score was dichotomized at the median and categorized as high vs low.

To evaluate the density of stained CD66b^+^ cells, the nucleated stained cells in each area were quantified and expressed as the number of cells per field at 200× magnification, as previously described.^17^, ^19^ Based on the median number of CD66b^+^ TANs in the training group, the patients were separated into high and low TAN groups.

MMR protein loss was defined as the absence of nuclear staining in tumor cells in the presence of positive nuclear staining in normal epithelial cells and lymphocytes. Tumors were categorized as having deficient MMR (dMMR) if the expression of at least one protein was lost and proficient MMR (pMMR) if all proteins were intact.

### 2.4 Nomogram construction

In the training cohort, survival curves for different variable values were generated using the Kaplan‐Meier estimates and were compared using the log‐rank test. Variables achieving significance at *P *<* *0.05 were entered into the multivariable analyses via the Cox regression model. Statistical analyses to identify independent prognostic factors were conducted in SPSS 17.0 for Windows (SPSS, Chicago, IL). Based on the multivariable Cox analysis results, two nomograms were developed by the rms packages using the R 3.2.4 (http://www.r-project.org). Variables were selected by the backward stepwise selection method in the Cox regression model.^37^

### 2.5 Nomogram validation and calibration

The model performance for predicting outcome was evaluated using the concordance index (C‐index),^4^ which is similar in concept to the area under a receiver operating characteristic curve. Calibration of the nomograms for 1‐, 3‐, and 5‐year DFS and OS was performed by comparing the predicted survival with the actual survival after bias correction. The performance of the nomograms was also validated in the validation cohort.

### 2.6 Clinical use

Decision curve analysis was conducted to assess the clinical usefulness of the model by quantifying the net benefits at different threshold probabilities.^38^, ^39^

### 2.7 Nomogram‐based risk group stratification

Using the X‐tile,^40^ the composite scoring of the nomograms was divided into three groups that accurately distinguished patients with a good, intermediate, and poor outcomes.

### 2.8 Statistical analysis

We compared two groups using the *t* test for continuous variables and the χ² test for categorical variables. Survival curves were generated according to the Kaplan‐Meier method and compared by the log‐rank test. Univariate and multivariate analyses were performed with the Cox proportional hazards model. All other statistical tests were performed using SPSS version 17.0 and R version 3.2.4 (http://www.r-project.org). Statistical significance was set at 2‐sided *P *<* *.05.

## 3 RESULTS

### 3.1 Clinicopathological correlations

**Figure 1:**
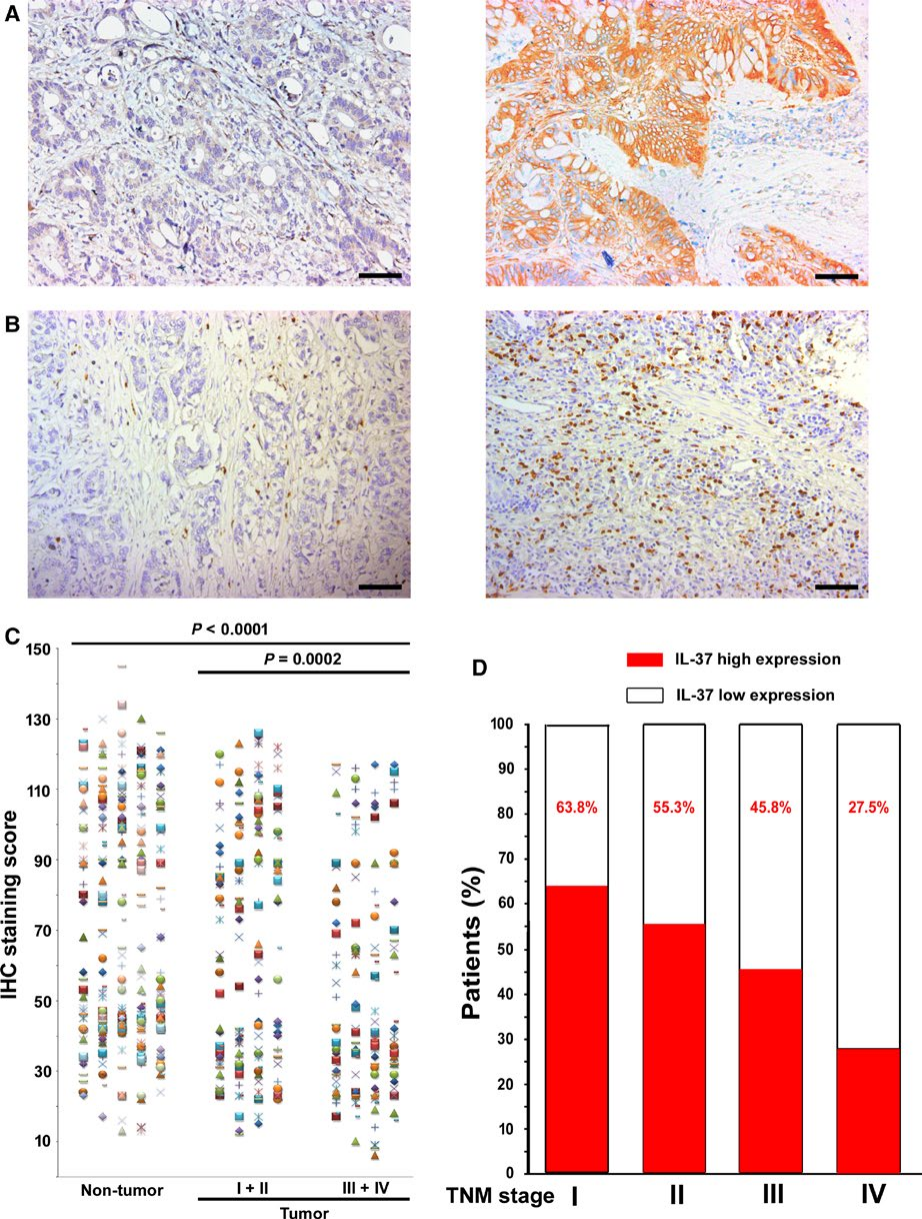
IL‐37 Expression in CRC Tissues. A and B, Representative IHC images of IL‐37 and CD66b expression in tumor tissue, left panel: low IL‐37and CD66b expression, right panel: high IL‐37 and CD66b expression. C, Scatter plots for IHC staining score in unpaired nontumor tissue (n = 337) and tumor tissue (n = 337) from the training cohort. *P* value was determined using the nonparametric Mann‐Whitney test. D, Percentage of patients with high intratumoral IL‐37 expression increased moderately with disease progression from TNM stage I‐IV (n = 582, data from the training and validation cohort). Scale bar, 100 μm

Tables 1 and S1‐S2 list the clinical characteristics of the patients and the clinicopathological correlations with IL‐37 expression, the CD66b^+^ TAN level, and MMR status in the training and validation cohorts. Specimens were available for analysis from 583 patients. The specific expression of cytoplasmic IL‐37 was observed in both nontumoral and intratumoral tissues, and the staining intensity was variable (Figure 1A). Compared with the nontumoral IL‐37 density in epithelial cells, the intratumoral IL‐37 expression in cancer cells was reduced (*P *<* *0.0001; Figure 1C). The expression of IL‐37 was much lower in advanced stage [stages I‐II (n = 185) vs stages III‐IV (n = 152), *P *=* *0.0002]. Furthermore, the percentage of patients with high IL‐37 expression reduced moderately along with disease progression from TNM stage I to IV (Figure 1D, Table 1). Lower IL‐37 expression was associated with a higher CD66b^+^ TAN level, a higher N stage, and a higher TNM stage (Figure S1, Table 1). The CD66b^+^ TAN level was associated with IL‐37 expression and MMR status (Table S1). pMMR was associated with less CD66b^+^ TAN infiltration and a higher T stage (Figure S1, Table S2).

**Table 1:** Clinical characteristics of patients according to IL‐37 in the training and validation cohorts

Variables	Training cohort (n = 337)				Validation cohort (n = 245)			
	N	Low IL‐37 (%)	High IL‐37 (%)	*P* value	N	Low IL‐37 (%)	High IL‐37 (%)	*P* value
Gender								
Male	208	105 (50.5%)	103 (49.5%)	0.877	145	77 (53.1%)	68 (46.9%)	0.212
Female	129	64 (49.6%)	65 (50.4%)		100	45 (45.0%)	55 (55.0%)	
Age (y)								
<60	176	91 (51.7%)	85 (48.3%)	0.55	125	61 (48.8%)	64 (51.2%)	0.75
≧60	161	78 (48.4%)	83 (51.6%)		120	61 (50.8%)	59 (49.2%)	
Tumor location								
Colon	155	80 (51.6%)	75 (48.4%)	0.62	108	54 (50.0%)	54 (50.0%)	0.955
Rectum	182	89 (48.9%)	93 (51.1%)		137	68 (49.6%)	69 (50.4%)	
Differentiation status								
Well	153	80 (52.3%)	73 (47.7%)	0.77	150	81 (54.0%)	69 (46.0%)	0.247
Moderate	140	68 (48.6%)	72 (51.4%)		73	31 (42.5%)	42 (57.5%)	
Poor and undifferentiated	44	21 (47.7%)	23 (52.3%)		22	10 (45.5%)	12 (54.5%)	
CEA								
Elevated	103	60 (58.3%)	43 (41.7%)	0.048	71	43 (60.6%)	28 (39.4%)	0.031
Normal	234	109 (46.6%)	125 (53.4%)		174	79 (45.4%)	95 (54.6%)	
CA199								
Elevated	58	27 (50.9%)	31 (49.1%)	0.547	42	26 (61.9%)	16 (38.1%)	0.085
Normal	279	142 (46.6%)	137 (53.4%)		203	96 (48.4%)	107 (51.6%)	
Depth of invasion								
T1	16	4 (25%)	12 (75%)	0.011	14	6 (42.9%)	8 (57.1%)	0.104
T2	46	20 (43.5%)	26 (56.5%)		45	18 (40.0%)	27 (60.0%)	
T3	206	99 (48.1%)	107 (51.9%)		118	55 (46.6%)	63 (53.4%)	
T4a	15	11 (73.3%)	4 (26.7%)		19	11 (57.9%)	8 (42.1%)	
T4b	54	35 (64.8%)	19 (35.2%)		49	32 (57.1%)	17 (42.9%)	
Lymph node metastasis								
N0	197	88 (44.7%)	109 (55.3%)	0.041	132	54 (40.9%)	78 (59.1%)	0.003
N1	99	55 (55.6%)	44 (44.4%)		78	43 (55.1%)	35 (44.9%)	
N2	41	26 (63.4%)	15 (36.6%)		35	25 (71.4%)	10 (28.6%)	
Metastasis								
M0	298	142 (47.7%)	156 (52.3%)	0.011	215	99 (46.0%)	116 (54.0%)	0.002
M1	39	27 (69.2%)	12 (30.8%)		30	23 (67.4%)	7 (32.6%)	
TNM stage								
I	52	20 (38.5%)	32 (61.5%)	0.023	42	14 (33.3%)	28 (66.7%)	0.001
II	133	62 (46.6%)	71 (53.4%)		82	34 (41.5%)	48 (58.5%)	
III	113	60 (53.1%)	53 (46.9%)		91	51 (56.0%)	40 (44.0%)	
IV	39	27 (69.2%)	12 (30.8%)		30	23 (76.7%)	7 (23.3%)	
CD66b cells/field (mean±SD)		70.1 ± 45.2	57.6 ± 34.2	0.004		65.5 ± 38.2	51.3 ± 34.1	0.003
CD66b								
Low	169	75 (44.4%)	94 (55.6%)	0.034	123	46 (37.4%)	77 (62.6%)	<0.0001
High	168	94 (56.0%)	74 (44.0%)		122	76 (62.3%)	46 (37.7%)	
MMR								
dMMR	63	19 (30.2%)	44 (69.8%)	0.0004	46	21 (45.7%)	25 (54.3%)	0.533
pMMR	274	150 (54.7%)	124 (45.3%)		199	101 (50.8%)	98 (49.2%)	

### 3.2 Prognostic value of IL‐37, CD66b, and MMR status

**Figure 2:**
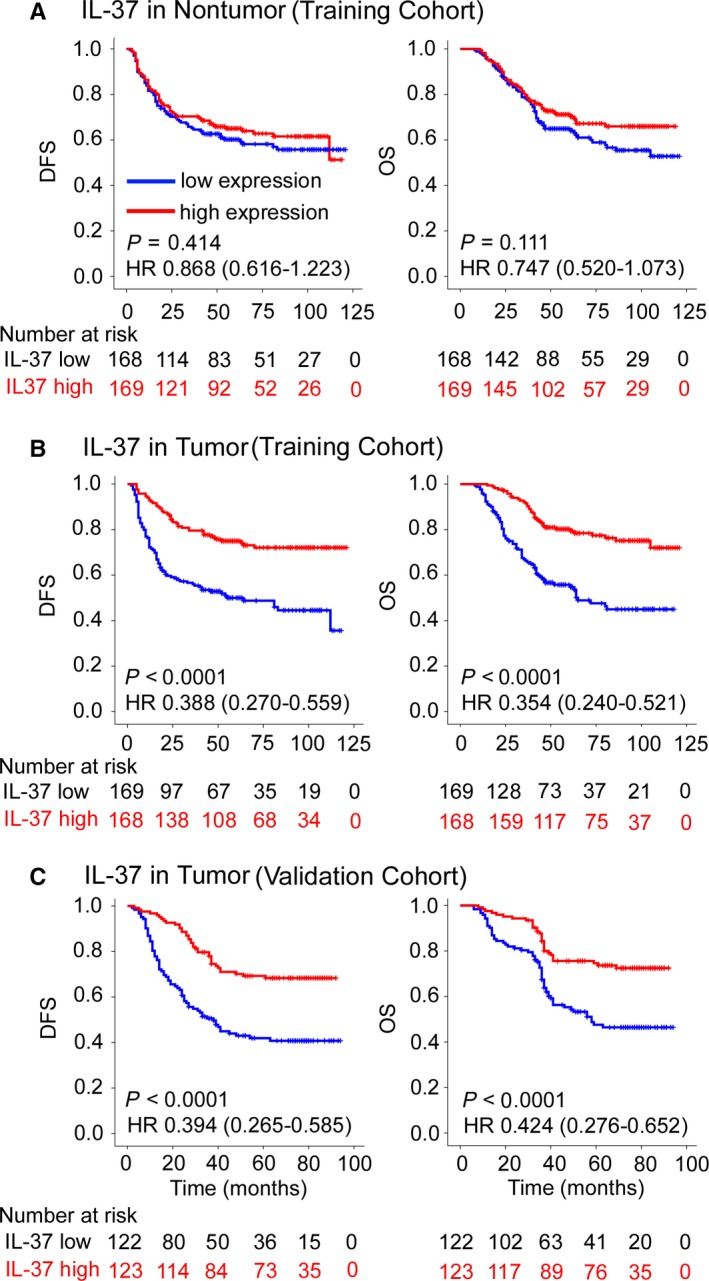
Kaplan‐Meier Survival Analysis of DFS and OS According to IL‐37 Expression in Nontumor (A) and Tumor (B, C) tissue of CRC Patients in the Training and Validation Cohorts. The left and right panels show the results from the training and validation cohorts, respectively

**Figure 3:**
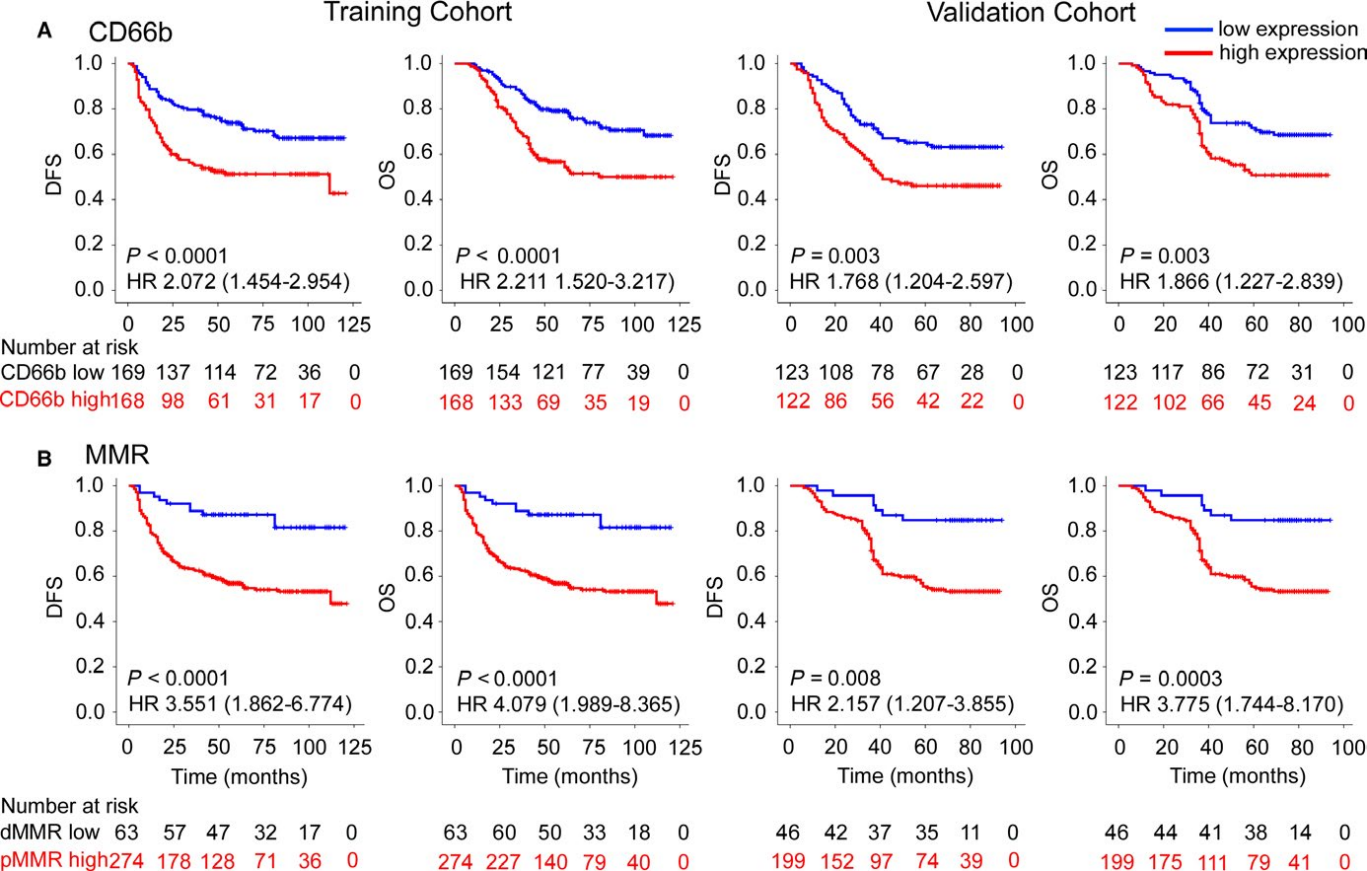
Kaplan‐Meier Survival Analysis of DFS and OS According to the CD66b Expression (A) and MMR Status (B) of CRC Patients in the Training and Validation Cohorts. The left and right panels show the results from the training and validation cohorts, respectively

In the training cohort, patients with high IL‐37 expression and fewer CD66b^+^ TANs showed statistically favorable DFS and OS (Figures 2 and 3). Patients with dMMR CRC had better clinical outcomes than patients with pMMR CRC (Figure 3). Similar results were observed in the validation cohort. The clinicopathological parameters for the prediction of DFS and OS were further investigated by univariate analysis with the Cox regression model. In the univariate and multivariate Cox regression analysis, the T stage, N stage, CEA level, IL‐37 expression, CD66b^+^ TAN level, and MMR status were significantly associated with DFS and OS (*P *<* *0.05, Tables 2 and S3‐S4). When stratified by clinicopathological variables, both IL‐37 and CD66b were still significant prognostic biomarkers (Figures S2‐S5).

**Table 2:** Multivariable Cox regression analysis in the training cohort

Variables	Disease‐free survival		Overall survival	
	HR (95% CI)	*P* value	HR (95% CI)	*P* value
CEA (ng*/*mL) (elevated vs normal)	1.617 (1.110‐2.354)	0.012	1.504 (1.013‐2.234)	0.043
Depth of invasion		0.0002		0.006
T4b	Reference		Reference	
T1	0.001 (0.0001‐999)	0.959	0.001 (0.0001‐999)	0.955
T2	0.299 (0.128‐0.701)	0.005	0.394 (0.158‐0.980)	0.045
T3	0.445 (0.272‐0.727)	0.001	0.557 (0.335‐0.926)	0.024
T4a	1.067 (0.537‐2.121)	0.854	1.233 (0.630‐2.414)	0.541
Lymph node metastasis		0.001		<0.0001
N0	Reference		Reference	
N1	2.225 (1.467‐3.375)	0.0002	2.290 (1.463‐3.582)	0.0003
N2	1.927 (1.134‐3.273)	0.015	3.088 (1.811‐5.264)	<0.0001
Metastasis (M1 vs M0)	1.838 (1.141‐2.963)	0.012	2.479 (1.521‐4.040)	0.0003
IL‐37 (high vs low)	0.588 (0.402‐0.859)	0.006	0.559 (0.374‐0.836)	0.005
CD66b (high vs low)	1.749 (1.193‐2.564)	0.004	2.028 (1.364‐3.014)	0.0005
MMR status (pMMR vs dMMR)	3.281 (1.645‐6.545)	0.001	3.770 (1.729‐8.218)	0.001

CEA, carcino‐embryonic antigen.

### 3.3 Development and validation of nomograms for predicting CRC prognosis

**Figure 4:**
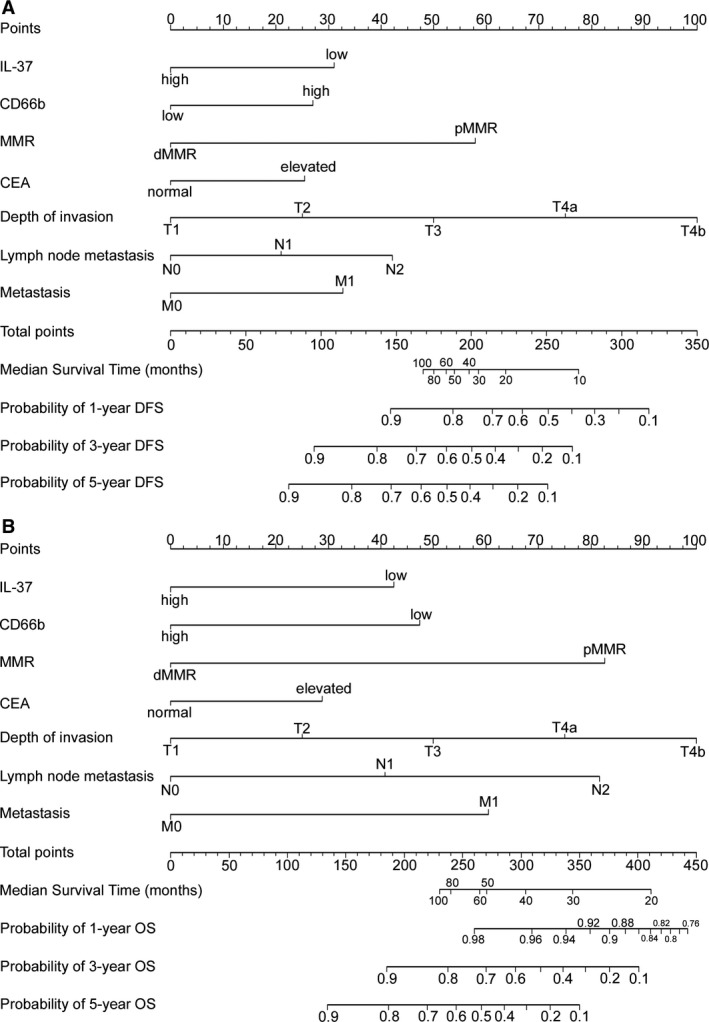
Nomogram for Predicting DFS and OS: Locate the Grade of the Patient on the Grade Axis and then Draw a Straight Line Upward to the Points Axis to Determine How Many Points Toward Survival the Patient Receives for her/his Grade. Repeat this process for the other axes, each time drawing a straight line upward toward the Points axis. Take the sum of the points received for each predictor and locate this sum on the Total Points axis. Draw a straight line down to the survival‐probability axis to find the patient's probability of surviving CRC

**Figure 5:**
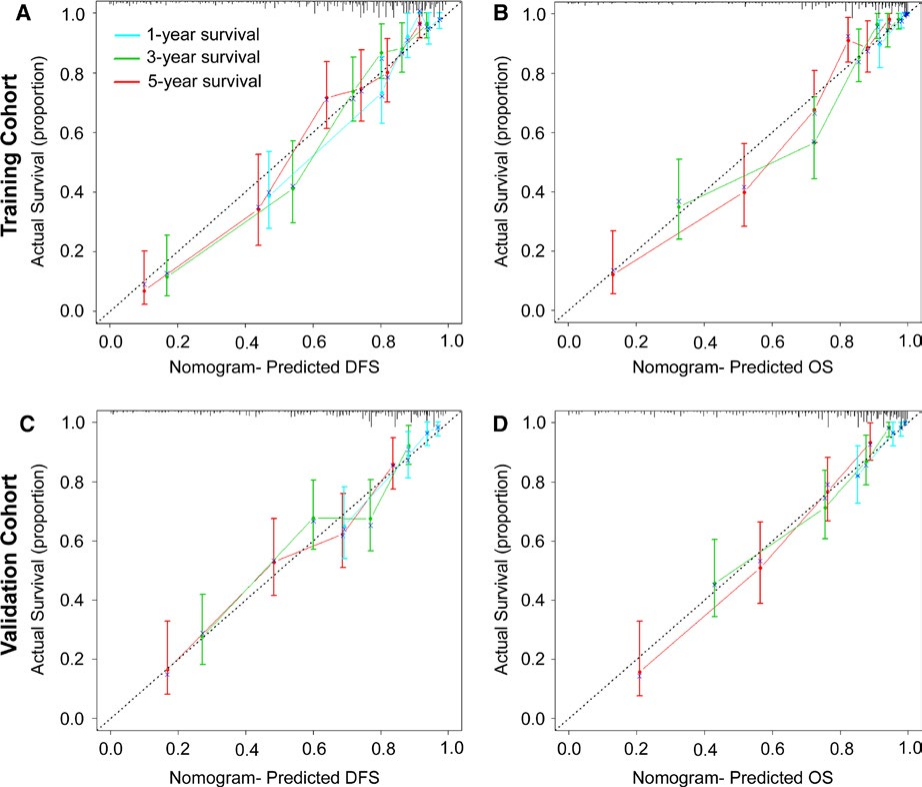
Calibration Curves for the Nomogram. The calibration curve for predicting patient DFS (A, C) and OS (B, D) at 1, 3, and 5 years in the training and validation cohorts. Nomogram‐predicted OS and DFS are plotted on the *x*‐axis, and actual OS and DFS are plotted on the *y*‐axis. The dotted line represents an ideal nomogram, and the solid line represents the current nomogram. The vertical bars represent 95% CIs, and the × symbols represent bootstrap‐corrected estimates

To predict DFS and OS, two nomograms were developed using the coefficients obtained from the multivariate Cox regression model (Figure 4A,B). To use the nomogram, first draw a vertical line up to the top Points row to assign points for each variable. Then, add the points from each variable together and drop a vertical line from the Total Points row to obtain the 1‐year, 3‐year, 5‐year, and median survival time (in months).^3^ The C‐indexes for the prediction of DFS and OS were 0.798 (95% confidence interval (CI) 0.764‐0.832) and 0.828 (0.796‐0.860), respectively, in the training cohort. Calibration curves for the two nomograms (Figure 5A,B) revealed no deviations from the reference line and no need for recalibration. The C‐indexes for the prediction of DFS and OS were 0.739 (0.696‐0.783) and 0.761 (0.715‐0.808), respectively, in the validation cohort. The calibration curves showed good agreement between the predicted and actual prognosis for 1‐, 3‐, and 5‐year DFS and OS (Figure 5C,D).

Moreover, we compared the discrimination of our nomograms with the TNM classification. The discrimination of our nomograms was superior to the TNM classification (C‐index, training cohort: DFS 0.741 (0.701‐0.780), OS 0.728 (0.689‐0.767), both *P *<* *0.0001; validation cohort: DFS 0.686 (0.638‐0.734), OS 0.697 (0.644‐0.749), both *P *<* *0.0001; respectively).

**Figure 6:**
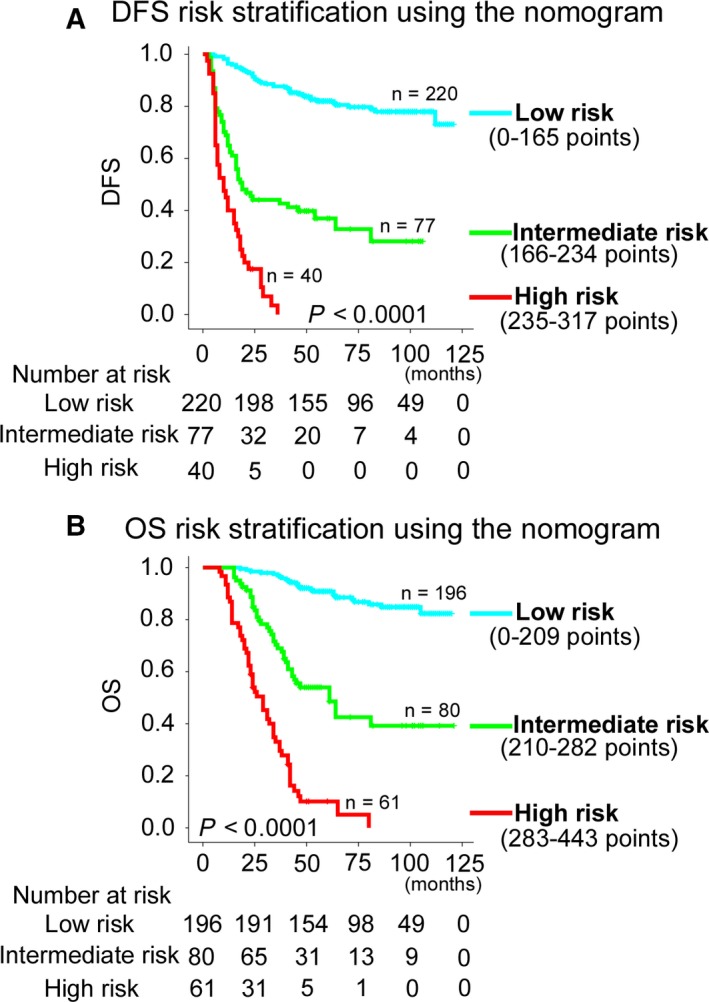
Kaplan‐Meier Survival Analysis of OS and DFS According to Three risk Groups in the Training Cohort. The entire population was divided in three subgroups according to the total number of points given by the nomograms. A, DFS nomogram. B, OS nomogram

**Figure 7:**
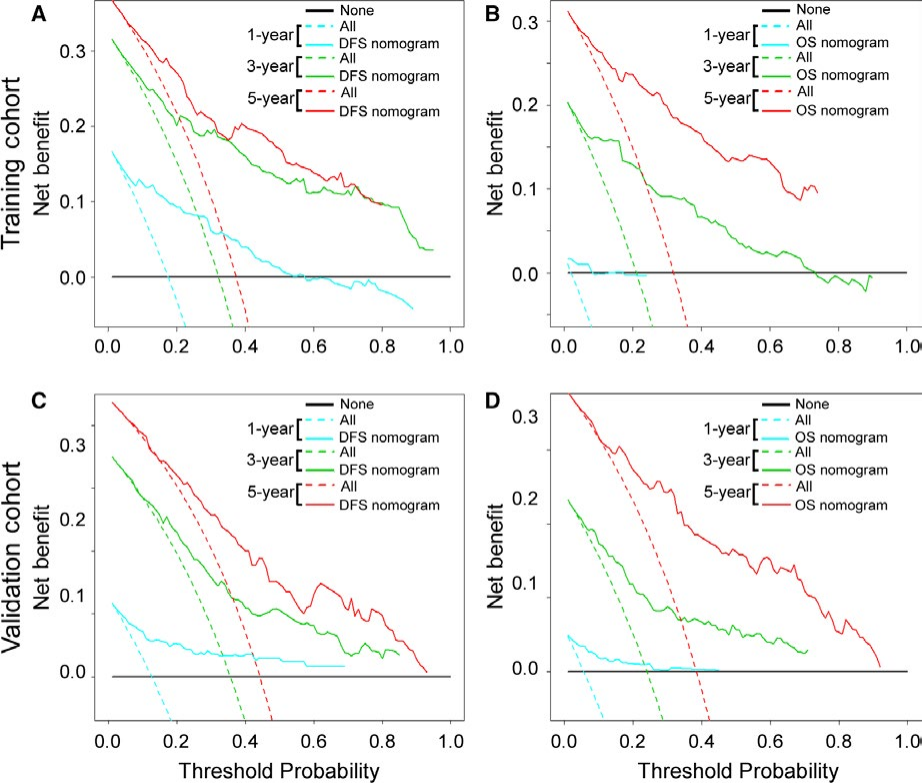
Decision Curve Analysis for the Two Nomograms in the Training and Validation Cohorts. The *y*‐axis measures the net benefit. The solid lines (yellow, blue and red) represent the nomogram. The dotted lines (blue, green and red) represent the assumption that all patients have 1‐, 3‐, or 5‐year survival, respectively. The thin black line represents the assumption that no patients have 1‐, 3‐, or 5‐year survival. The net benefit was calculated by subtracting the proportion of all patients who are false‐positive from the proportion who are true‐positive, weighted by the relative harm of forgoing treatment compared with the negative consequences of an unnecessary treatment.^38^, ^39^ (A, C): DFS nomogram. (B, D): OS nomogram

Using the X‐tile, the composite scoring of the nomograms was divided into three groups that accurately distinguished between patients with a good, intermediate, and poor outcomes (Figures 6 and S6). The three risk groups were validated in the validation cohort (Figure S7). Furthermore, we analyzed subgroups of patients in different stages. The three risk groups significantly distinguished patients with different prognoses at stage II, III, or IV (Figures S8‐S10). The decision curve analysis of the nomograms is shown in Figure 7. The decision curve showed that if the threshold probability of a patient or doctor was >10%, using the two nomograms to predict 1‐, 3‐, 5‐year DFS and OS added more benefit than either the treat‐all‐patients scheme or the treat‐none scheme.^38^ Within this range, the net benefit was comparable to several overlaps on the basis of the nomograms.^41^

## 4 DISCUSSION

IL‐37 is the 7th factor (IL‐1F7) of interleukin‐1 family (IL‐1F), and it is widely expressed in human organs and tissues. IL‐37 has been identified as a natural suppressor of innate inflammatory and immune responses.^8^, ^12^ Recent studies have indicated that IL‐37 plays a protective role in tumor progression in mouse fibrosarcoma,^9^ human lung cancer,^14^, ^42^ hepatocellular carcinoma,^13^ breast cancer,^43^ cervical cancer,^44^ and renal cell carcinoma.^45^ Zhang et al^15^ found that IL‐37 inhibits colon cancer progression via β‐catenin suppression and that a lack of IL‐37 is associated with poor survival. However, the prognostic value of IL‐37 was shown using IHC in 186 colon cancer patients from a single center. Despite this knowledge, the prognostic roles of IL‐37 in CRC have not yet been determined and still require validation. In this study, we sought to investigate the prognostic significance of IL‐37 in two large cohorts of CRC patients (n = 582). Using immunohistochemical staining, we found that IL‐37 expression is reduced in CRC tissues and that the percentage of patients with high IL‐37 expression decreases with increasing TNM stage (Figure 1D), suggests that IL‐37 may play an inhibitory role in the development of CRC. Furthermore, we demonstrated that high IL‐37 expression is an independent factor of a better prognosis.

Neutrophils play an important role in the acute phase of inflammation and in resistance against invading pathogens. Neutrophils have been ignored for a long time as a component of the inflammatory tumor microenvironment. Accumulating evidence shows that TANs are an important component of tumor‐related inflammation. Furthermore, recent evidence shows that TANs are endowed with considerable plasticity basing on environmental cues. In CRC, the presence and significance of TANs have been the subjects of conflicting reports.^20^, ^22^, ^24^ In this present study, we set out to rigorously evaluate the prognostic significance of TANs in CRC. One reason for the controversy is that there is still no uniform opinion on the method to distinguish neutrophils in CRC tissues. Compared with MPO and CD15, two other markers known to stain neutrophils in tissues, CD66b is a specific marker for neutrophils in CRC tissues. In this study, we demonstrated that a high CD66b^+^ TAN level is associated with a poor prognosis. CXCL8 mRNA and protein levels have been found to be significantly upregulated upon IL‐37 downregulation in colon epithelial cells, and CXCL8 can selectively recruit neutrophils.^10^ This suggests that IL‐37 might inhibit the recruitment of neutrophils, which is in consistent with our results that low IL‐37 expression was associated with a high CD66b^+^ TAN level in CRC.

IL‐37 is a natural suppressor of immune responses. Previous study also showed that IL‐37 mediated anti‐tumor immune responses through recruiting NK cells to tumor microenvironment in hepatocellular carcinoma.^13^ However, Ge et al^14^ found no change in TILs frequencies in tumors over‐expressing IL‐37, suggesting that IL‐37 might not affect the anti‐tumor immune responses in vivo. Also, IL‐37‐over‐expressing tumors had decreased CD34 level, which suggested an inhibited tumor angiogenesis. Furthermore, neutrophils could largely secret matrix metalloproteinase‐9 to stimulate proangiogenic activity of cancer cells.^19^ Our results showed high IL‐37 expression was associated with low CD66b^+^ TAN infiltration. Thus, IL‐37 may inhibit tumor angiogenesis through recruiting neutrophils, which needs further exploration.

Tumor MMR deficiency might be detected as MSI or the loss of MMR protein expression by IHC. We evaluated the prognostic role of the MMR status determined by IHC in two large cohorts of CRC patients. In the present study, dMMR patients whose tumors exhibited the loss of at least one MMR protein (MLH1, MSH2, MSH6, or PMS2) had a better prognosis. An unexpected finding was an association between dMMR status and an elevated CD66b^+^ TAN level. Of interest, consistent with recent work by Park et al^6^, dMMR status was associated with an elevated circulating neutrophil count.

Extensive literature has suggested that TANs are of clinical importance in cancer.^16^, ^17^, ^18^ Li et al^19^ showed that TANs are an independent and unfavorable factor in the prognosis of gastric cancer patients. However, Sun et al^26^ found that gastric cancer patients with high CD66b^+^ TAN levels gain a survival benefit and are prone to improved OS from postoperative adjuvant chemotherapy. In contrast, low TAN levels identified a subgroup of muscle invasive bladder cancer (MIBC) patients who appeared to benefit from adjuvant chemotherapy.^16^ In CRC patients, Galdiero et al^22^ revealed that a higher TAN density was associated with a better response to 5‐FU‐based chemotherapy. And this also indicates that neutrophils show different functions under different tumor microenvironments. The relationship between TANs and chemotherapy efficacy needs further study. Although the use of MMR status to predict the outcomes of adjuvant chemotherapy is still controversial, the results from previous trials are very promising and indicate that 5‐FU is beneficial for treating dMMR CRC.^28^, ^32^, ^33^ A large multicenter AGEO study indicated that high‐risk stage II dMMR colon cancer tended to have better outcomes with oxaliplatin‐based adjuvant chemotherapy than with surgery alone.^34^ However, before MMR status can be used as a predictive biomarker in clinical practice, its value must be proven in large, high‐powered prospective trials. Zhang et al^15^ showed that IL‐37 might sensitize colon cancer to chemotherapy in mice. Future studies will investigate the relationship of IL‐37 with chemotherapy in CRC and investigate the potential mechanism.

The genomes of cancers deficient in mismatch repair (MMR) contain exceptionally high numbers of somatic mutations.^28^ According to a phase II study, dMMR CRCs were sensitive to immune checkpoint blockade with anti‐PD‐1 antibodies.^29^ Recent evidence supports the hypothesis that the large proportion of mutant neoantigens in dMMR cancers make them sensitive to immune checkpoint blockade, regardless of the cancers’ tissue of origin.^27^ IL‐37 has shown anti‐inflammatory and immune suppression effects. IL‐37 contributes to the immunosuppressive property of human regulatory T cells, and its antitumor activity was identified by the recruitment of more natural killer cells in hepatocellular carcinoma.^11^, ^13^ In addition, we found that low IL‐37 expression and dMMR status are associated with high CD66b^+^ TAN levels in CRC. These findings suggest that IL‐37 may be useful for tumor immunotherapy.

The TNM staging system is the most popularly used system for predicting outcomes in CRC patients. Nevertheless, patients with the same stage could show different genetic, cellular, and clinicopathological characteristics and outcomes.^46^ To offer a more individualized tool, nomograms have been constructed to assess a mass of significant predictors to better predict the outcomes of individuals. Improved prediction of individual prognosis could be useful for counseling patients, personalizing treatment, and scheduling patient follow‐ups.^3^, ^4^ Although several CRC nomograms are available, no particular nomogram has been widely clinically used.^3^, ^4^ In the study, we constructed and validated the two nomograms integrating the IL‐37 expression determined by IHC, CD66b^+^ TAN level, MMR status, T stage, N stage, M stage, and CEA level to improve the accuracy of prediction. The nomograms could be utilized to better estimate an individual patient's probability of 1‐, 3‐, and 5‐year DFS and OS. The C‐index and calibration curves were used to validated the nomograms. The C‐index for DFS and OS was satisfactory (0.836 (0.804‐0.868) and 0.841 (0.808‐0.874), respectively, in the training cohort). Furthermore, the nomograms performed well with good calibration. Compared with previous studies, our nomograms included three prognosis biomarkers (IL‐37, CD66b^+^ TAN level and MMR status) that extremely improved the accuracy.

In addition, the improved outcome estimates measured using the nomograms might help to identify patients with a high risk of poor outcome with known TNM stages, and also to facilitate the choice of treatment. Current guidelines recommend adjuvant chemotherapy for high‐risk stage II patients. The risk of a poor outcome or relapse in stage II disease can be clinically identified by the following: fewer than 12 lymph nodes examined; lymphatic/vascular invasion; poorly differentiated histology (exclusive to those that are MSI‐H); perineural invasion; bowel obstruction; localized perforation; and close, indeterminate, or positive margins.^4^ However, a proportion of stage II and III patients without an increased risk of relapse based on current clinical factors still do relapse. Therefore, these clinicopathological variables cannot be used to accurately identify high‐risk patients who may benefit from chemotherapy.^4^ Accordingly, the two nomograms, which incorporate multiple prognostic parameters into the current staging system, may assist in identifying patients who are likely to benefit from chemotherapy.

This study also has some limitations. First, the study is a retrospective study relying uniquely on a single‐center data. Validation from external cohorts is necessary for the prognostic value of IL‐37 and the widespread use of the nomograms as the basis of treatment recommendations. Further studies of the biological function of IL‐37 expression in cell lines and animal models should be performed in the future. Other prognostic and/or predictive factors may be integrated to enhance the accuracy of the nomograms. In addition, the application of the nomograms requires the results of several IHC‐based analyses and pathological variables that are only available after surgery, that is, pT stage and pN stage. Hence, the nomograms will have limited influence on alternative treatments prior to surgery, including neoadjuvant chemotherapy.

In conclusion, our results demonstrate that IL‐37 expression and the CD66b^+^ TAN level are independent predictors of patient outcome. Low IL‐37 expression and CD66b^+^ TAN levels were associated with a poor prognosis, and dMMR patients had better clinical outcomes. Two nomograms integrating these three markers and four clinicopathological variables were developed for predicting 1‐, 3‐, and 5‐year DFS and OS probabilities after curative resection. The nomograms performed well with good discrimination and calibration, identifying the model as a simple and easy tool for estimating the survival of individual patients with CRC.

## CONFLICT OF INTEREST

No potential conflicts of interest were disclosed.
