# Supplementary material for: Prognostic significance of nomograms integrating IL‐37 expression, neutrophil level, and MMR status in patients with colorectal cancer
Source: Cancer Med. 2018 Jul 13;7(8):3682–94. doi: 10.1002/cam4.1663 (PMC6089143; doi:10.1002/cam4.1663)
Supplement: Supplementary file 1 [file CAM4-7-3682-s001.pdf]

Supplementary Materials

Supplementary materials includes ten supplementary figures, and four supplementary tables.

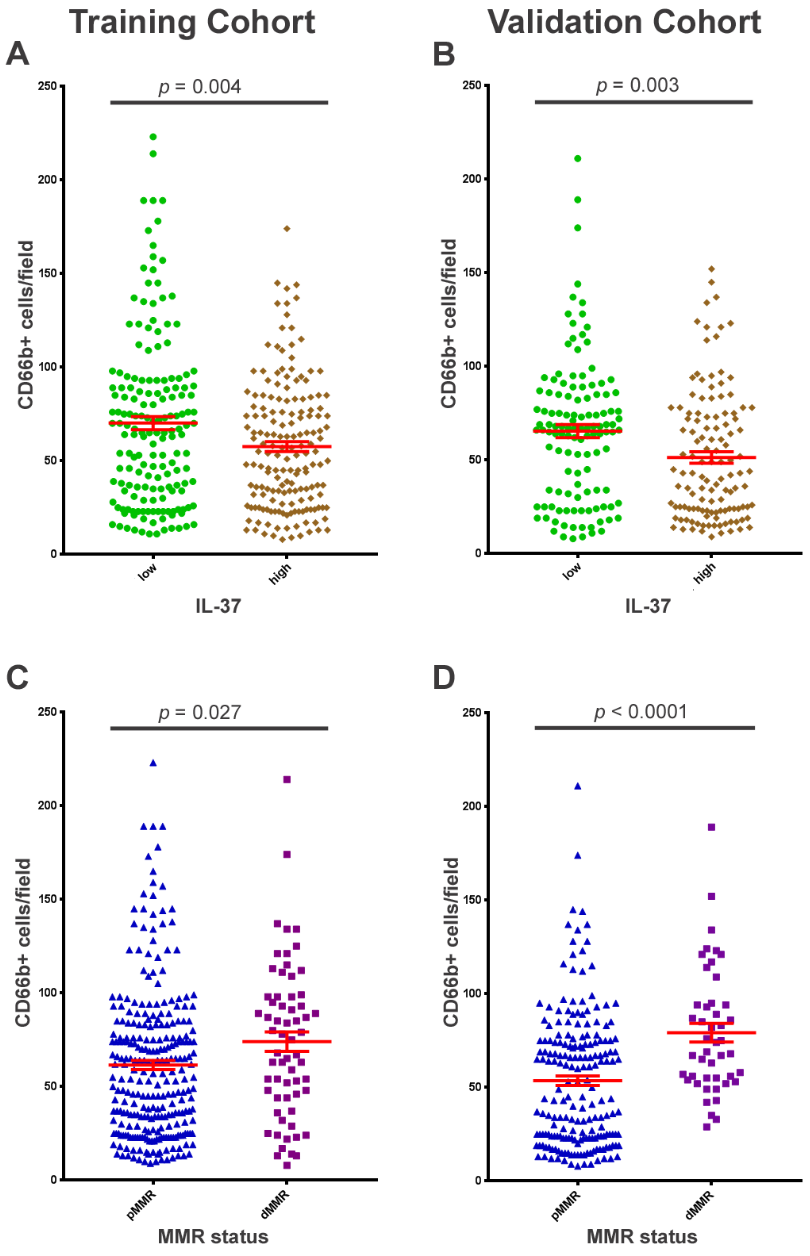

Figure S1: Scatter plots for CD66b+ neutrophils per field according to IL-37 expression and MMR status in the training and validation cohorts. (A, B): IL-37 expression, (C, D): MMR status. The left panel shows the results from the training cohort, and the right panel shows the results from the validation cohort.

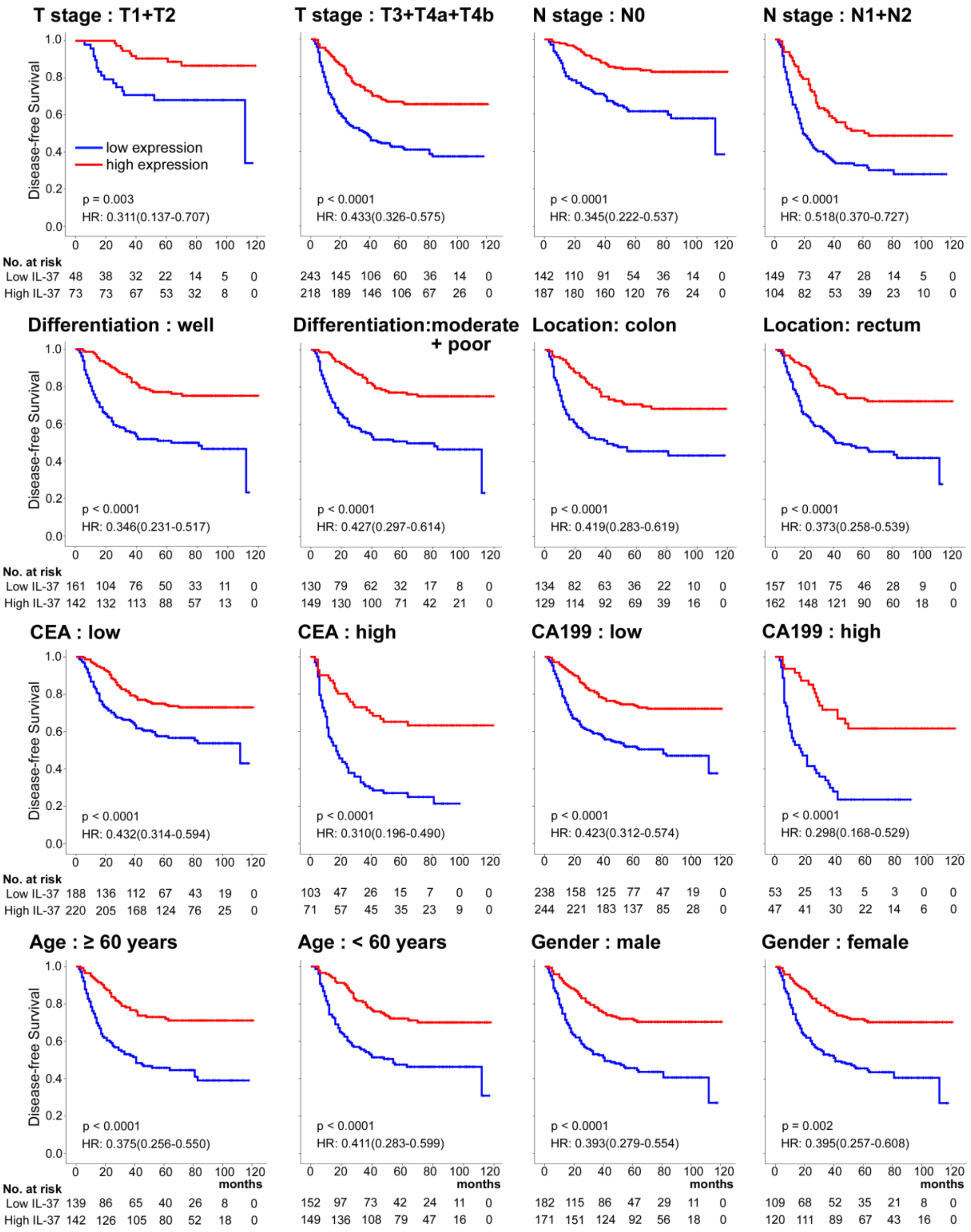

Figure S2: Kaplan-Meier survival analysis of OS for all 582 patients with colorectal cancer according to the IL-37 expression stratified by clinicopathological risk factors. *P*-values were calculated by log-rank test.

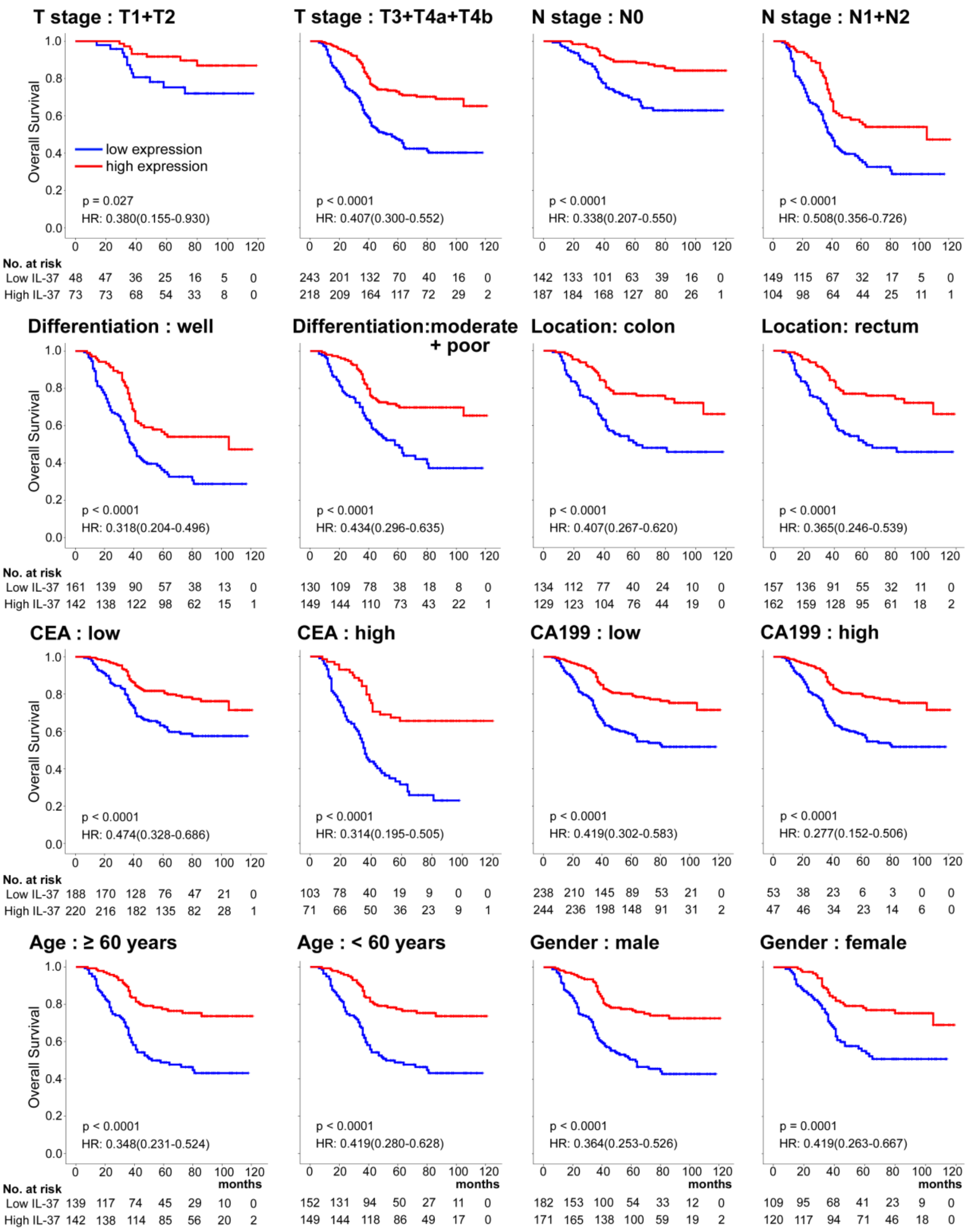

Figure S3: Kaplan-Meier survival analysis of disease-free survival (DFS) and overall survival (OS) according to CD66b expression (A) and MMR status (B) of CRC patients in the training cohort and validation cohort. The left panel shows the results from the training cohort, and the right panel shows the results from the validation cohort.

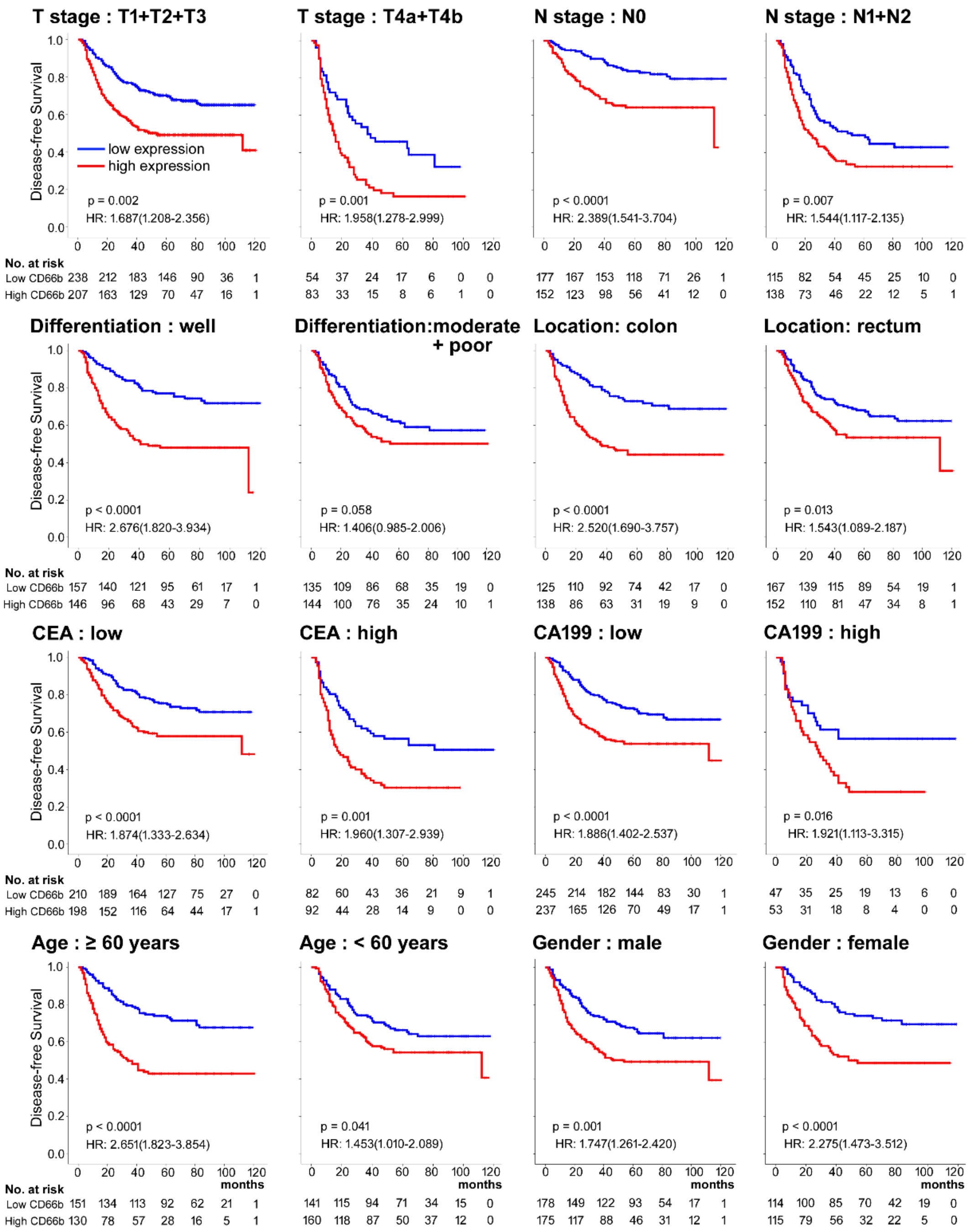

Figure S4: Kaplan-Meier survival analysis of DFS for all 582 patients with CRC according to CD66b expression stratified by clinicopathological risk factors. P-values were calculated by the log-rank test.

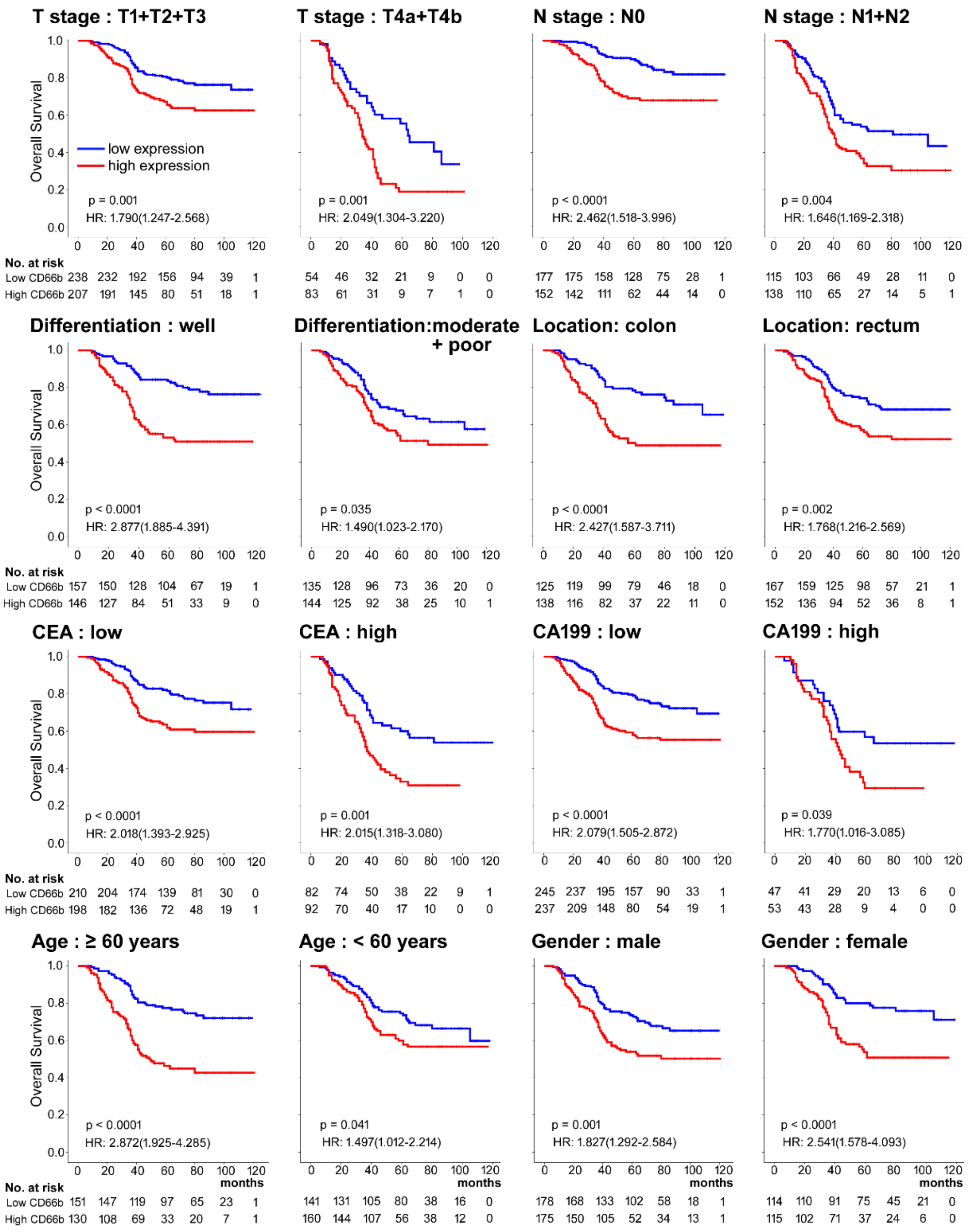

Figure S5: Kaplan-Meier survival analysis of OS for all 582 patients with CRC according to CD66b expression stratified by clinicopathological risk factors. P-values were calculated by the log-rank test.

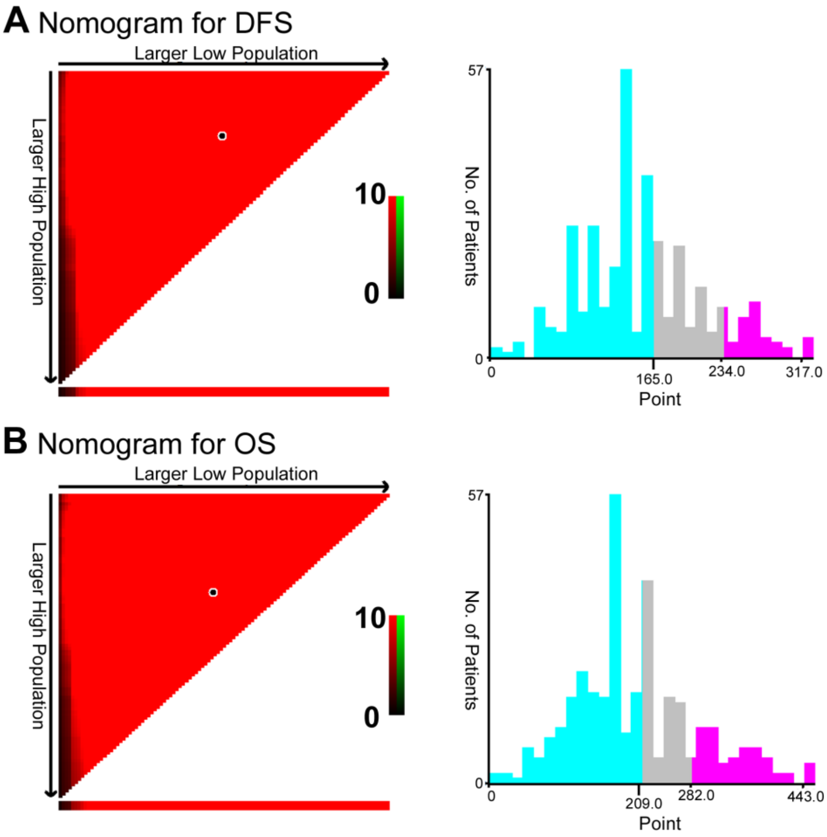

Figure S6: X-tile plots of the two nomograms and the points of the nomograms.

Coloration of the plot represents the strength of the association at each division ranging from low (dark, black) to high (bright, red, or green). Red represents the inverse association between the expression levels and survival of the feature, whereas green represents a direct association. (A): DFS nomogram, (B): OS nomogram.

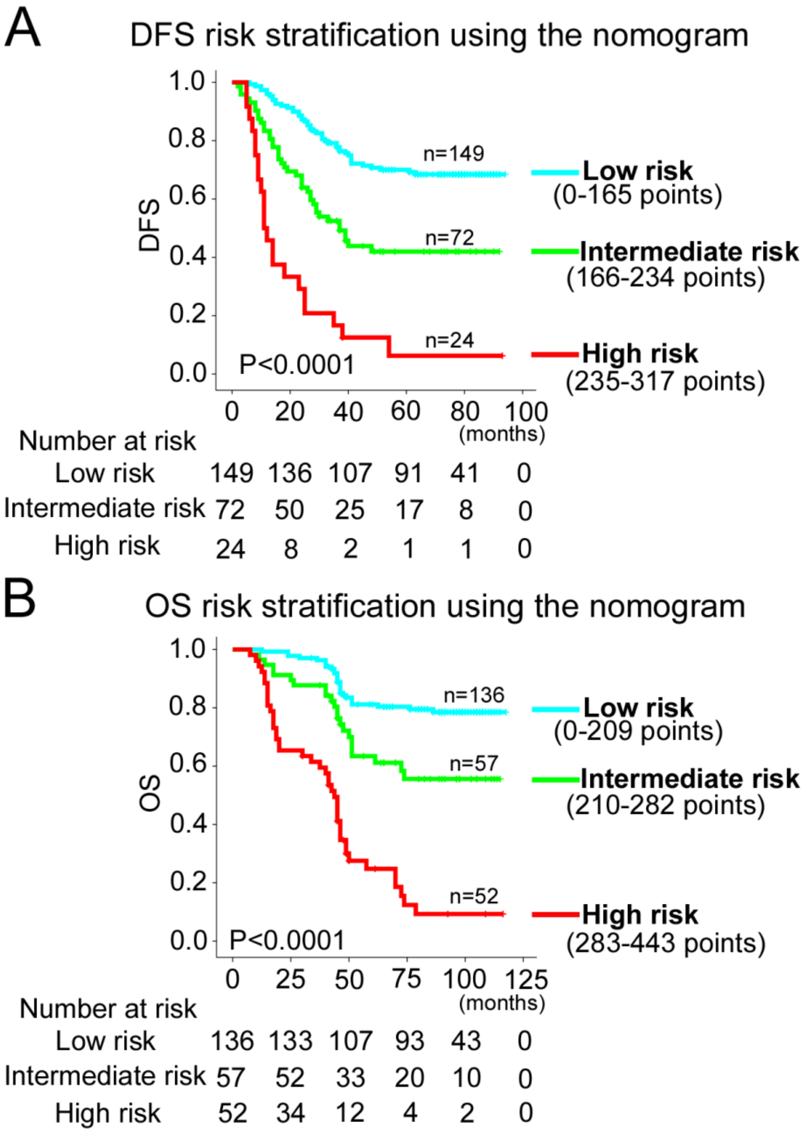

Figure S7: Kaplan-Meier survival analysis of DFS and OS according to the three risk groups in the validation cohort. The entire population was divided into 3 subgroups according to the total number of points given by the nomograms. (A): DFS nomogram, and (B): OS nomogram.

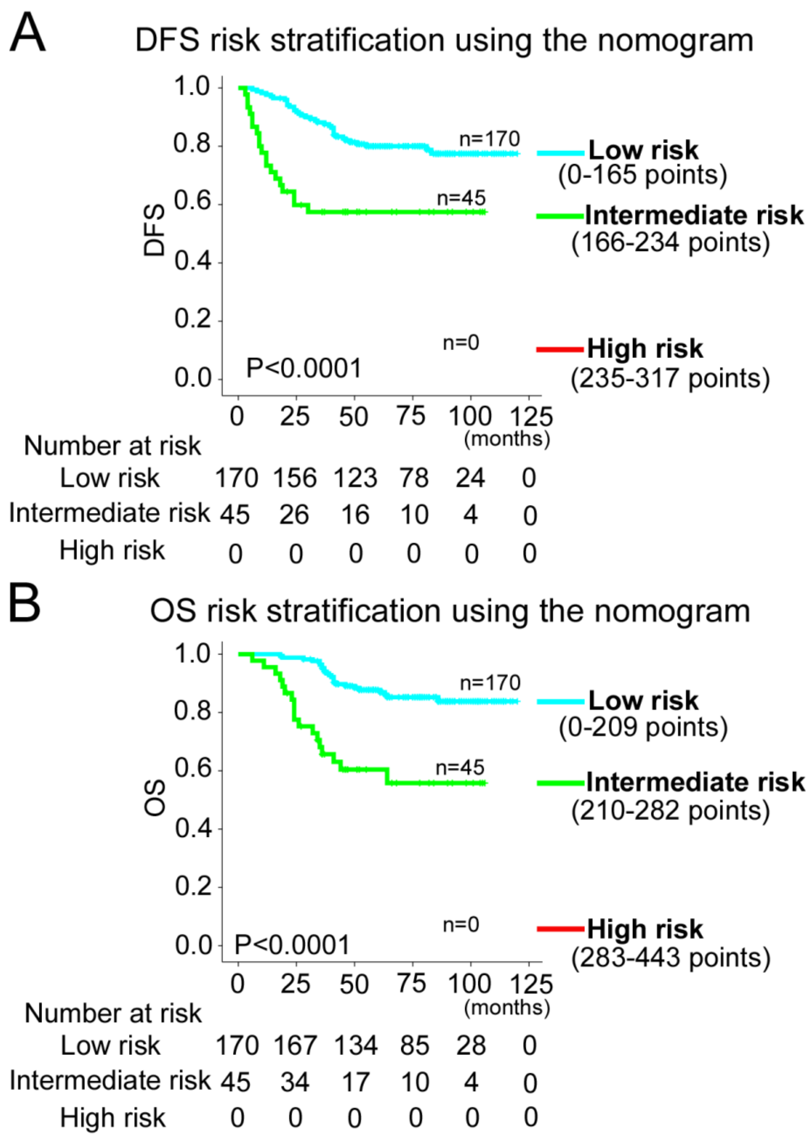

Figure S8: Kaplan-Meier survival analysis of DFS and OS according to the three risk groups in stage II CRC patients. The entire population was divided into 3 subgroups according to the total number of points given by the nomograms. (A): DFS nomogram, and (B): OS nomogram.

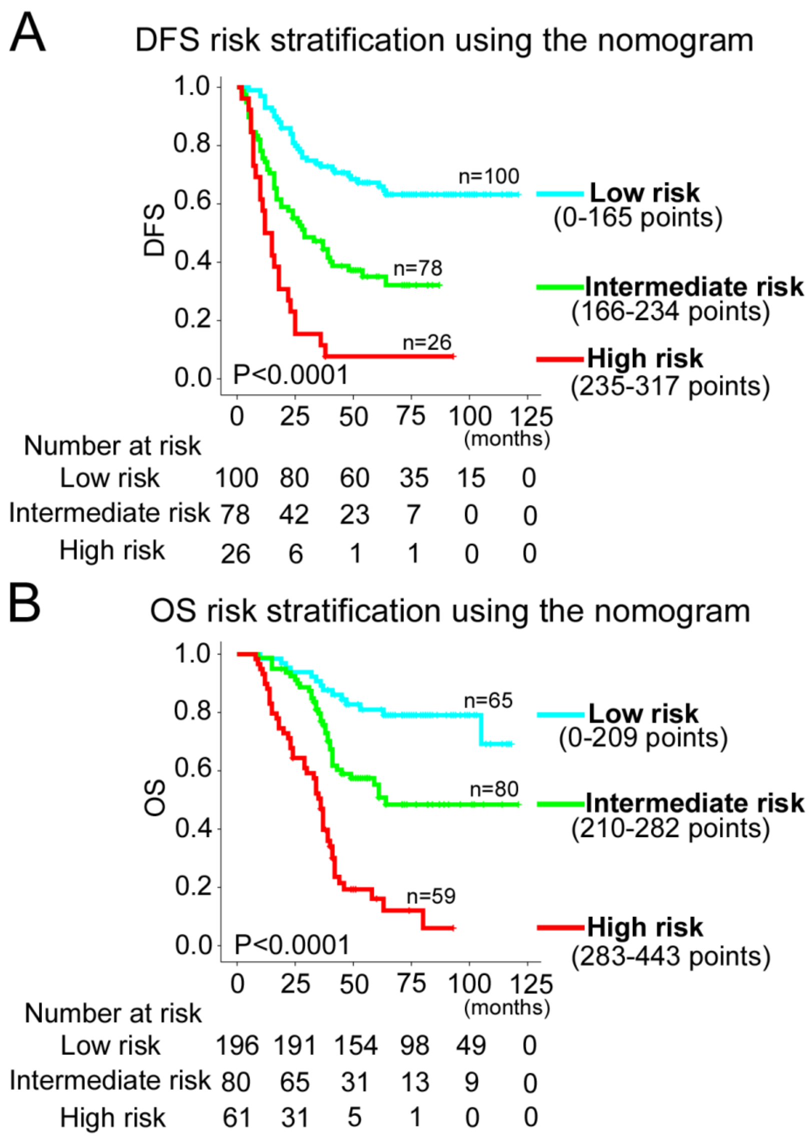

Figure S9: Kaplan-Meier survival analysis of DFS and OS according to the three risk groups in stage III CRC patients. The entire population was divided into 3 subgroups according to the total number of points given by the nomograms. (A): DFS nomogram, and (B): OS nomogram.

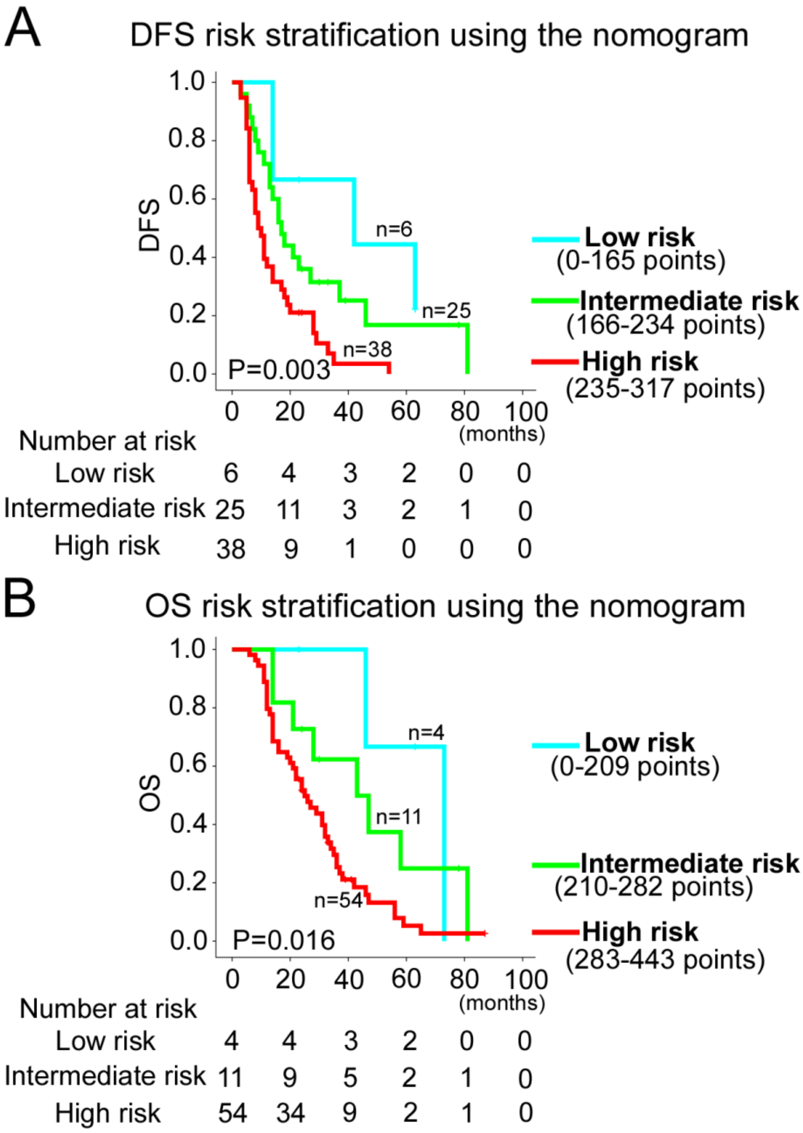

Figure S10: Kaplan-Meier survival analysis of DFS and OS according to the three risk groups in stage IV CRC patients. The entire population was divided into 3 subgroups according to the total number of points given by the nomograms. (A): DFS nomogram, and (B): OS nomogram.

Table S1. Clinical characteristics of patients according to CD66b in the training and validation cohorts.

**Variables**	**Training cohort (n = 337)**				**Validation cohort (n =245)**			
	N	low IL-37 (%)	high IL-37 (%)	p value	N	low IL-37 (%)	high IL-37 (%)	p value
**Gender**				0.546				0.641
Male	208	107(51.4%)	101(48.6%)		145	71(49.0%)	74(51.0%)	
Female	129	62(48.1%)	67(51.9%)		100	52(52.0%)	48(48.0%)	
**Age(years)**				0.172				0.337
＜60	176	82(46.6%)	94(53.4%)		125	59(47.2%)	66(52.8%)	
≧60	161	87(54.0%)	74(46.0%)		120	64(53.3%)	56(46.7%)	
**Tumor location**				0.056				0.647
Colon	155	69(44.5%)	86(55.5%)		108	56(51.9%)	52(48.1%)	
Rectum	182	100(54.9%)	82(45.1%)		137	67(48.9%)	70(51.1%)	
**Differentiation status**				0.567				0.394
Well	153	80(52.3%)	73(47.7%)		150	77(51.3%)	73(48.7%)	
Moderate	140	70(50.0%)	70(50.0%)		73	38(52.1%)	35(47.9%)	
Poor and undifferentiated	44	19(43.2%)	25(56.8%)		22	8(36.4%)	14(63.6%)	
**CEA**				0.530				0.456
Elevated	103	49(47.6%)	54(52.4%)		71	33(46.5%)	38(53.5%)	
Nomal	234	120(51.3%)	114(48.7%)		174	90(51.7%)	84(48.3%)	
**CA199**				0.547				0.713
Elevated	58	27(50.9%)	31(49.1%)		42	20(47.6%)	22(52.4%)	
Nomal	279	142(46.6%)	137(53.4%)		203	103(50.7%)	100(49.3%)	
**Depth of invasion**				0.003				0.53
T1	16	12(75%)	4(25%)		14	5(35.7%)	9(64.3%)	
T2	46	26(56.5%)	20(43.5%)		45	24(53.3%)	21(46.7%)	
T3	206	107(51.9%)	99(48.1%)		118	64(54.2%)	54(45.8%)	
T4a	15	9(60.0%)	6(40.0%)		19	8(42.1%)	11(57.9%)	
T4b	54	15(27.8%)	39(72.2%)		49	22(44.9%)	27(55.1%)	
**Lymph node metastasis**				0.186				0.216
N0	197	106(53.8%)	91(46.2%)		132	71(53.8%)	61(46.2%)	
N1	99	47(59.5%)	52(40.5%)		78	39(50.0%)	39(50.0%)	
N2	41	16(39.0%)	25(61.0%)		35	13(37.1%)	22(62.9%)	
**Metastasis**				0.384				0.233
M0	298	152(51.0%)	146(49.0%)		215	111(51.6%)	104(48.4%)	
M1	39	17(43.6%)	22(56.4%)		30	12(40.0%)	18(60.0%)	
**TNM stage**				0.099				0.343
I	52	34(65.4%)	18(34.6%)		42	23(54.8%)	19(45.2%)	
II	133	66(49.6%)	67(50.4%)		82	46(56.1%)	36(43.9%)	
III	113	52(46.0%)	61(54.0%)		91	42(46.2%)	49(53.8%)	
IV	39	17(43.6%)	22(56.4%)		30	12(40.0%)	18(60.0%)	
**CD66b cells/field (mean±SD)**		32.2±13.9	95.8±32.7	<0.0001		30.2±21.7	86.8±25.4	<0.0001
**IL-37**				0.034				<0.0001

low	169	75(44.4%)	94(55.6%)		122	46(37.7%)	76(62.3%)	
high	168	94(56.0%)	74(44.0%)		123	77(62.6%)	46(37.4%)	
**MMR**				0.034				0.02
dMMR	63	24(38.1%)	39(61.9%)		46	16(34.8%)	30(65.2%)	
pMMR	274	145(52.9%)	129(47.1%)		199	107(53.8%)	92(46.2%)	

Table S2. Clinical characteristics of patients according to MMR status in the training and validation cohorts.

Variables	Training cohort (n = 337)				Validation cohort (n =245)			
	N	dMMR (%)	pMMR (%)	p value	N	dMMR (%)	pMMR (%)	p value
**Gender**				0.543				0.356
Male	208	41(19.7%)	167(80.3%)		145	30(20.7%)	115(79.3%)	
Female	129	22(17.1%)	107(82.9%)		100	16(16.0%)	84(84.0%)	
**Age(years)**				0.759				0.248
＜60	176	34(19.3%)	142(80.7%)		125	27(21.6%)	98(78.4%)	
≧60	161	29(18.0%)	132(82.0%)		120	19(15.8%)	101(84.2%)	
**Tumor location**				0.397				0.674
Colon	155	32(20.6%)	123(79.4%)		108	19(17.6%)	89(82.4%)	
Rectum	182	31(17.0%)	151(83.0%)		137	27(19.7%)	110(80.3%)	
**Differentiation status**				0.408				0.194
Well	153	33(21.6%)	120(78.4%)		150	31(20.7%)	119(79.3%)	
Moderate	140	24(17.1%)	116(82.9%)		73	14(19.2%)	59(80.8%)	
Poor and undifferentiated	44	6(13.6%)	38(86.4%)		22	1(4.5%)	21(95.5%)	
**CEA**				0.197				0.118
Elevated	103	15(14.6%)	88(85.4%)		71	9(12.7%)	62(87.3%)	
Nomal	234	48 (20.5%)	186(79.5%)		174	37(21.3%)	137(78.7%)	
**CA199**				0.755				0.092
Elevated	58	10(17.2%)	48(82.8%)		42	4(9.5%)	38(90.5%)	
Nomal	279	53(19.0%)	226(81.0%)		203	42(20.7%)	161(79.3%)	
**Depth of invasion**				0.003				0.011
T1	16	5(31.2%)	11(68.8%)		14	7(50.0%)	7(50.0%)	
T2	46	12(26.1%)	34(73.9%)		45	10(22.2%)	35(77.8%)	
T3	206	39(18.9%)	167(81.1%)		118	21(17.8%)	97(82.2%)	
T4a	15	2(13.3%)	13(86.7%)		19	4(21.1%)	15(78.9%)	
T4b	54	5(9.3%)	49(90.7%)		49	4(8.2%)	45(91.8%)	
**Lymph node metastasis**				0.020				0.191
N0	197	45(22.8%)	152(77.2%)		132	29(22.0%)	103(78.0%)	
N1	99	16(16.2%)	83(83.8%)		78	14(17.9%)	64(82.1%)	
N2	41	2(4.9%)	39(95.1%)		35	3(8.6%)	32(91.4%)	
**Metastasis**				0.151				0.070
M0	298	59(19.8%)	239(80.2%)		215	44(20.5%)	171(79.5%)	
M1	39	4(10.3%)	35(89.7%)		30	2(6.7%)	28(93.3%)	
**TNM stage**				0.066				0.109
I	52	12(23.1%)	40(76.9%)		42	12(28.6%)	30(71.4%)	
II	133	32(24.1%)	101(75.9%)		82	17(20.7%)	65(79.3%)	
III	113	15(13.3%)	98(86.7%)		91	15(16.5%)	76(83.5%)	
IV	39	4(10.3%)	35(89.7%)		30	2(6.7%)	28(93.3%)	
**CD66b cells/field(mean±SD)**		74.1±41.1	61.5±40.1	0.027		79.2±33.5	53.6±36.0	<0.0001
**IL-37**				0.0004				0.533
low	169	19(11.2%)	150(88.8%)		122	21(17.2%)	101(82.8%)	

high	168	44(26.2%)	124(73.8%)		123	25(20.3%)	98(79.7%)	
**CD66b**				0.034				0.02
low	63	24(14.2%)	145(85.8%)		46	16(13.0%)	107 (87.0%)	
high	274	39(23.2%)	129(76.8%)		199	30(24.6%)	92(75.4%)	

Table S3. Univariate association of IL-37, CD66b, MMR, clinicopathological characteristics with OS and DFS in the training cohort.

**Variables**	**Disease-free survival**		**Overall survival**	
	HR (95% CI)	p value	HR (95% CI)	p value
Age(years) (≥60 vs. <60)	1.005 (0.714-1.417)	0.976	1.019 (0.711-1.461)	0.917
Gender (male vs. female)	1.024 (0.721-1.455)	0.894	1.020 (0.706-1.476)	0.915
Tumor location (colon vs.rectum)	1.058 (0.750-1.492)	0.747	1.040 (0.725-1.491)	0.832
Differentiation status	1.341 (1.048-1.717)	0.020	1.457 (1.128-1.881)	0.004
CEA(ng/ml) (elevated vs. nomal)	2.215 (1.565-3.134)	<0.0001	2.354 (1.638-3.382)	<0.0001
CA199(ng/ml) (elevated vs. nomal)	1.804 (1.199-2.714)	0.005	2.061 (1.361-3.120)	0.001
Depth of invasion	2.230 (1.886-2.636)	<0.0001	2.296 (1.928-2.735)	<0.0001
Lymph node metastasis	2.488 (3.690-8.230)	<0.0001	2.838 (2.246-3.586)	<0.0001
Metastasis (M1 vs. M0)	5.510 (6.437-15.941)	<0.0001	6.638 (4.396-10.025)	<0.0001
IL-37 (high vs. low)	0.388 (0.270-0.559)	<0.0001	0.354 (0.240-0.521)	<0.0001
CD66b (high vs. low)	2.072 (1.454-2.954)	<0.0001	2.211 (1.520-3.217)	<0.0001
MMR status (pMMR vs. dMMR)	3.551 (1.862-6.774)	<0.0001	4.079 (1.989-8.365)	<0.0001

CEA: carcino-embryonic antigen.

Table S4. Univariate association of IL-37, CD66b, MMR, clinicopathological characteristics with OS and DFS in the validation cohort.

Variables	Disease-free survival		Overall survival	
	HR (95% CI)	p value	HR (95% CI)	p value
Age(years) (≥60 vs. <60)	1.133 (0.777-1.652)	0.517	1.248 (0.829-1.881)	0.288
Gender (male vs. female)	1.411 (0.951-2.095)	0.088	1.530 (0.993-2.358)	0.054
Tumor location (colon vs.rectum)	1.170 (0.802-1.707)	0.416	1.124 (0.746-1.692)	0.577
Differentiation status	1.482 (1.128-1.948)	0.005	1.506 (1.120-2.025)	0.007
CEA(ng/ml) (elevated vs. nomal)	2.396 (1.631-3.518)	<0.0001	2.451 (1.621-3.705)	<0.0001
CA199(ng/ml) (elevated vs. nomal)	1.896 (1.224-2.930)	0.004	1.897 (1.190-3.023)	0.007
Depth of invasion	1.477 (1.251-1.743)	<0.0001	1.501 (1.256-1.795)	<0.0001
Lymph node metastasis	2.076 (1.629-2.647)	<0.0001	2.239 (1.724-2.908)	<0.0001
Metastasis (M1 vs. M0)	5.003 (3.156-7.929)	<0.0001	5.123 (3.191-8.223)	<0.0001
IL-37 (high vs. low)	0.394 (0.265-0.585)	<0.0001	0.424 (0.276-0.652)	<0.0001
CD66b (high vs. low)	1.768 (1.204-2.597)	0.004	1.866 (1.227-2.839)	0.004
MMR status (pMMR vs. dMMR)	2.157 (1.207-3.855)	0.009	3.775 (1.744-8.170)	0.001

CEA: carcino-embryonic antigen.
